# The role of environmental contextual cues in sequence learning: evidence from a virtual maze context

**DOI:** 10.1007/s00426-023-01868-y

**Published:** 2023-08-19

**Authors:** Iring Koch, Otmar Bock

**Affiliations:** 1 Institute of Psychology, RWTH Aachen University, Jägerstr. 17-19, 52056 Aachen, Germany; 2 Institute of Exercise Training and Sport Informatics, Deutsche Sporthochschule Köln/German Sport University, Cologne, Germany

## Abstract

Studies on sequence learning usually focus on single, isolated stimuli that are presented sequentially. For example, in the serial reaction time (RT) task, stimuli are either presented in a predictable sequence or in a random sequence, and better performance with the predictable sequence is taken as evidence for sequence-specific learning. Yet, little is known about the role of environmental context cues in sequence learning. If the target stimuli are embedded in a meaningful context, would this facilitate learning by providing helpful contextual associations or would it hinder learning by adding distracting stimuli? This question was examined in two studies. A pilot study compared sequence learning in a virtual maze with a horizontal vs. vertical maze context, in which arrow stimuli guide spatial lever movement responses that resulted in a corresponding virtual transport on the screen. The results showed only overall somewhat better performance with the vertical maze compared to the horizontal maze, but general practice effects and sequence-specific learning effects were the same for both contexts. The main study compared sequence learning with a maze context to sequence learning of arrows without a maze context. The results showed significantly better learning with maze context than without context. These data suggest that the maze context facilitated sequence learning by inducing a meaningful spatial representation (“mental map”) similar to that formed in wayfinding.

## Introduction

Our ability to speed up responses to predictable sequences of events is of practical relevance in many real-life situations such as computer use, piano playing or car driving. To study sequence learning processes, serial reaction time (SRT) tasks have evolved as a powerful experimental tool (Nissen & Bullemer, 1987; see Schwarb & Schumacher, 2012, for a review). In the SRT task, stimuli and responses are often spatial in nature, such as different stimulus locations on a computer screen and manual button presses on a keyboard, respectively. For example, Nissen & Bullemer (1987) presented their stimuli in one of four horizontally aligned positions on the screen, and the response keys were mapped to the screen locations in a spatially compatible manner. These authors found that RTs became gradually shorter with a predictable and cyclic 10-element stimulus sequence, compared to a control group responding to a random sequence of stimuli (see also Hoffmann & Koch, 1997), demonstrating sequence learning. Because participants are often unaware of the stimulus sequence; this RT effect represents a performance measure of learning that is potentially dissociable from verbal, “explicit” measures of learning. Therefore, the SRT task has been termed an “implicit learning” task (Abrahamse et al., 2010; Dienes & Berry, 1997; Shanks & St. John, 1994, for discussion).

Later studies developed methodological refinements to examine several interrelated questions. One question refers to how explicit awareness of the sequence can be measured, leading to the development of more sophisticated explicit learning measures (such as cued recall and recognition tests), with a focus on whether learning can be implicit at all (see Shanks & St. John, 1994; Esser et al., 2022, for discussion). Another question refers to what kind of internal representation is actually formed in sequence learning. For example, when using a 10-element sequence on four screen locations, like in Nissen & Bullemer’s (1987) original work, the frequencies of each individual location differ from each other, so that particularly implicit learning could be driven by simple frequency learning (Shanks et al., 1994; see Reed & Johnson, 1994). Therefore, so-called second order conditional [SOC] sequences have been introduced, in which the pairwise transitions in the sequence are fully balanced, so that observed learning effects must be sequence-specific at least at the level of pairs of stimuli and/or responses (see Reed & Johnson, 1994, for a review and empirical analysis). Moreover, the assessment of the RT performance score of learning is now typically no longer done with a between-group comparison, but rather with a random transfer sequence after practice with a training sequence, which represents a statistically more powerful comparison. Yet another question addresses the roles of stimulus type (i.e., spatial location vs. symbolic stimuli) or of stimulus and response modality (e.g., Blotenberg et al., 2018; Goschke & Bolte, 2012; Keele et al., 2003; Kemeny & Meier, 2016; Koch & Hoffmann, 2000; Riedel & Burton, 2006; Zirngibl & Koch, 2002; see Koch et al., 2020, for discussion).

In general, sequence learning has so far been investigated mostly in isolation, using classical laboratory paradigms. In everyday life, however, sequence learning is typically embedded in a rich and meaningful behavioral context, which could modify performance substantially. Indeed, previous research has documented that behavioral context is a powerful modifier of various cognitive and motor skills. This has been shown by comparing locomotion in the laboratory versus in a community park (Bock & Beurskens, 2010), manual grasping as an isolated act versus as part of a computer game (Bock & Züll, 2013) or as part of grocery shopping (Kim & Bock, 2019), and decision making, concentration (Moskaliuk et al., 2017), perceptual speed, episodic memory, working memory, spatial skills and reasoning (Verhaeghen et al., 2012) in standardized tests versus in the workplace. Given this evidence, it appears likely that behavioral context would modify sequence learning as well.

On the one hand, the presence of a context could degrade sequential performance since the processing of contextual cues might compete with sequence learning for common cognitive resources, just as secondary tasks were found to compete with sequence learning (overview e.g. in Schumacher & Schwarb, 2009). On the other hand, the presence of a context could enhance performance by associating the to-be-learned sequence to an internal representation of the context, just as semantic and episodic memory is enhanced by contextual associations (Godden & Baddeley, 1975; Yonelinas et al., 2019). A popular example of elaboration by association with contextual information is the mnemonic technique called “method of loci,” where items are mentally “attached” to locations along a familiar route (overview e.g. in Legge et al., 2012).

To find out whether sequence learning is degraded or enhanced by a meaningful context, we designed a serial RT task where participants saw recurring sequences of arrows, and had to move their hand as fast as possible in the direction indicated by each arrow. They executed this task either in isolation or in a route-following context. In the latter case, they were asked to repeatedly follow a prescribed route through a virtual maze. When they reached an intersection, an arrow appeared to indicate the direction in which the route continued. Thus, the recurring sequence of arrows was embedded in a recurring sequence of directions to take along a prescribed route. We expected that participants form a mental representation of the route, like a “cognitive map” (O’Keefe & Nadel, 1978; Tolman, 1948), and that this map affects learning of the arrow sequence.

In a pilot study, we first established our experimental sequence-learning paradigm as a SRT task embedded in a maze context. To this end, we compared participants’ RTs in a horizontally oriented maze to those in a vertically oriented maze. The sequence of arrows and manual responses was the same in both learning conditions, but the behavioral context was different. We assessed learning in two ways. First, we examined general practice benefits across twenty practice runs of 12 arrows each. Then, we tested sequence-specific learning by comparing performance in the last practice run (Run 20) with a new transfer sequence in the following run (Run 21), so that any sequence-specific learning can no longer be applied and any performance decrement would indicate learning beyond unspecific practice with the task and response device.

For the pilot study, we reasoned that it might be easier to form a mental representation of the route in a horizontal rather than in a vertical plane, since everyday life offers little opportunity for route following in a vertical plane (see Bock et al., 2020, for a discussion). It has indeed been documented, in studies of navigation in buildings, that the mental representation of objects is more accurate for objects located on the same level of a building rather than on different levels (Montello & Pick, 1993), and for objects encountered along a mainly horizontal rather than a mainly vertical route (Thibault et al., 2013; Zwergal et al., 2016), which led to the hypothesis that multistorey buildings are mentally represented as a stack of horizontal layers rather than as a genuinely three-dimensional structure (Jeffrey et al., 2013). We therefore examined whether sequence learning would benefit from a route-following context more if the route is oriented horizontally rather than vertically. Yet, to anticipate the results of the pilot study, we only found a small general performance benefit of the vertical maze over the horizontal maze but no significant differences in sequence-specific learning.

Based on the pilot study, the main study compared participants’ learning performance in a maze context (oriented horizontally or vertically) to that without a maze context. Again, the sequence of arrows and responses was the same in both learning conditions, but the behavioral context was different. We hypothesized that learning performance would be better with the maze context than without that context, even though the sequence of stimuli and responses is the same.

## Methods

### Participants and general design

In the pilot study, 24 healthy young persons (21.1 ± 3.0 years of age, 21 female, 3 male) participated. Half of the participants were tested first with a horizontal maze for context, and then with a vertical maze for context; the other half were tested in the reverse order (i.e., counterbalanced order of maze orientations). In the main study, 32 healthy young persons (22.4 ± 3.1 years of age, 24 female, 8 male) participated. Half of them were tested first with a maze context and then without a maze context, and the other half were tested in the reverse order. Because the pilot study produced no learning differences between horizontal vs. vertical maze contexts (see below), the maze context in the main study was horizontal for half of the participants and vertical for the other half, and data were collapsed across maze orientations for analysis. The studies were carried out in accordance with the declaration of Helsinki (World Medical Association, 2013). Informed consent was given by all participants before testing began.

### Stimuli and task

**Fig. 1:**
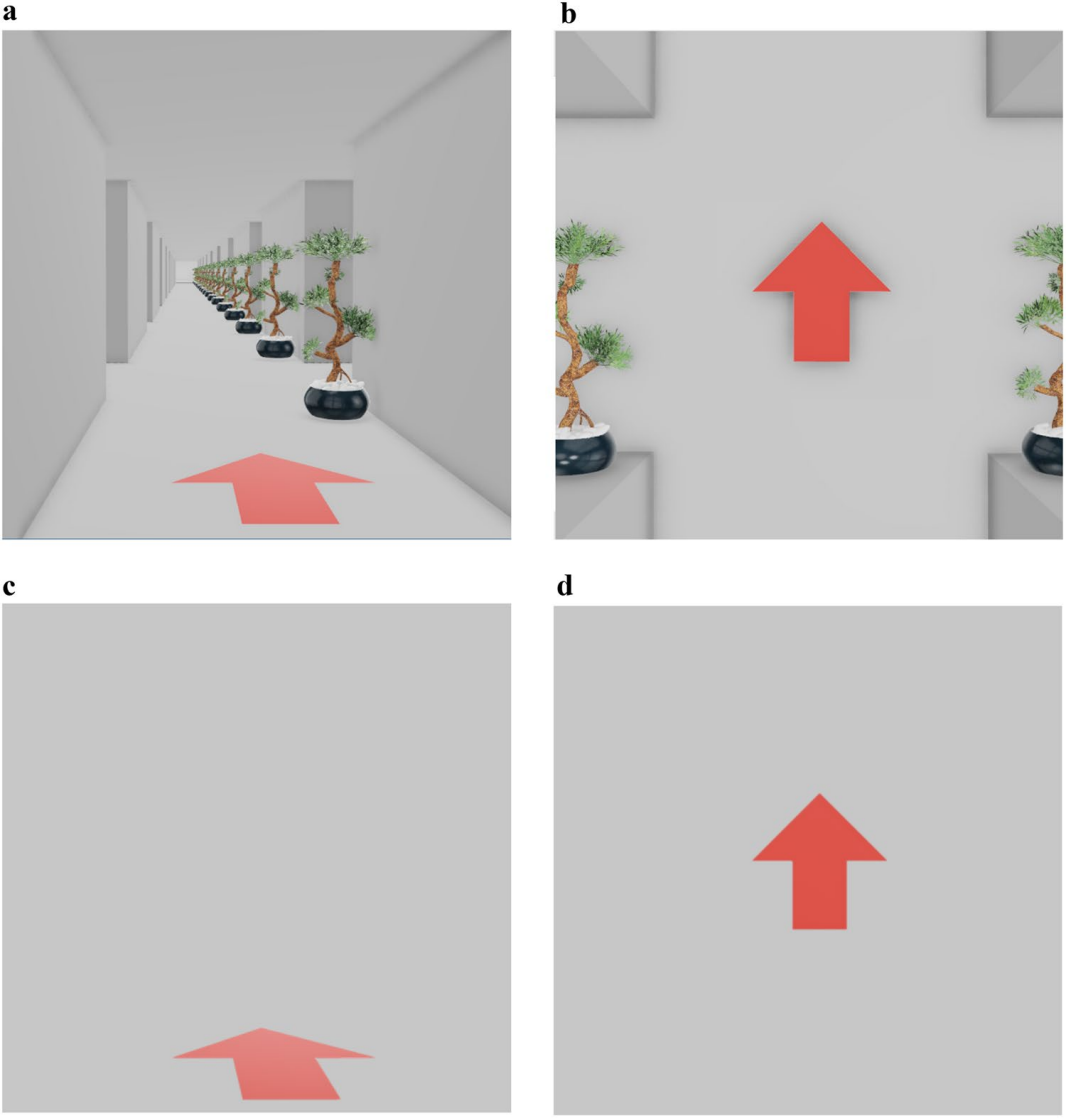
Examples of the monitor display in our studies

Participants sat in front of a 19″ computer monitor on which a sequence of 12 red arrows was displayed. The sequence was presented either in a horizontal plane, with arrows pointing forwards, backwards, left- or rightwards, or in a vertical plane, with arrows pointing upwards, downwards, left- or rightwards. The sequence was displayed either at the intersections of a maze that was oriented in the same plane as the arrow sequence (i.e., vertically or horizontally) or against a featureless background. The images that constituted the maze were implemented by the architecture design software pCon planner. Images were quadratic with a side length of 870 pixels and displayed at the screen center in their actual size with the remaining screen surface in black. The whole experiment was run in PsychoPy, version v2021.2.3 (Peirce et al., 2019). Figure 1 illustrates a forward-pointing arrow in a horizontal maze (Fig. 1a), a forward-pointing arrow without a maze context (Fig. 1c), an upward-pointing arrow in a vertical maze (Fig. 1b), and an upward-pointing arrow without a maze context (Fig. 1d). Note that the arrows for the horizontal maze were presented near the bottom of the image because this is where people usually look when walking, but we expected no consequences of this for the learning process.

The walls, floors, and ceilings of the maze were of uniform grey. A pair of potted plants was placed at each intersection to emphasize maze orientation (cf. Fig. 1); the color, shape, size, and location of those plants was the same at all intersections. Thus, all intersections in a maze looked exactly alike, except that the arrows could point in different directions.

The participants’ task was always to move their hand in the direction of the arrow. They grasped a 2 × 2 cm plate at the end of a handle with their dominant hand, using their thumb and the middle segment of their index finger, while their forearm rested on a height-adjustable, padded support. Once an arrow appeared on the monitor, they moved the plate in the corresponding direction, thus engaging a four-way switch that registered their responses. Switch and plate were positioned either to allow movements in a horizontal plane (forwards, backwards, left-, and rightwards), or to allow movements in a vertical plane, (upwards, downwards, left-, and rightwards). This enabled us to vary the plane in which the arrows were presented independently from the plane in which responses were produced, and thus to ensure that any observed effects of the stimulus plane are not confounded by effects of the response plane, or of the congruence between both planes. If stimulus and response planes were different, upward and downward arrows were mapped onto forward and backward responses, respectively, while forward and backward arrows were mapped onto upward and downward responses, respectively. We considered this a spatially compatible mapping since the same mapping is used for movements of a computer mouse and a mouse cursor.

After each response, participants were passively transported in the selected direction at a constant speed. We decided to implement purely translational transport, since rotations would not be equivalent in a horizontal and in a vertical maze. To familiarize participants with this mode of transport, they were shown the model of a maze intersection with a pair of miniature potted plants in their proper locations. The model was shown in the same orientation as in the upcoming experimental runs, and the experimenter used a Lego^®^ figure to demonstrate transportation. If the maze was oriented horizontally, the experimenter held the figure with its feet towards the earth and moved it without body turns forwards, backwards, left- and rightwards across an intersection. If the maze was oriented vertically, the experimenter held the figure with its feet towards the earth—not towards the maze floor—and moved it without body turns upwards, downwards, left- and rightwards across an intersection.

### Procedure

At the beginning of the study, after obtaining informed consent, the task was explained to the participants and that they should respond as quickly as possible to the arrows. After that, each trial started with the presentation of an arrow. If participants responded by moving their hand correctly, they were passively transported in the selected direction at a constant speed. For example, if a rightward arrow was presented against a homogeneous background and participants moved their hand rightwards, they saw the arrow move linearly towards the left edge of the monitor and then disappear; if a rightward arrow was presented at a maze intersection and participants moved their hand rightwards, they saw arrow and maze move towards the left edge of the monitor and disappear. In either case, they then saw the lateral wall of the maze corridor. Subsequently, they saw the next intersection—without an arrow—appear from the right edge of the monitor and move towards the center. This passive transport was simulated by successively displaying 19 images, each of which showing one part of the movement, for 120 ms each. After these 2.3 s of movement, the next arrow was displayed.

We chose a response-stimulus interval of 2.3 s to ensure a realistic “transportation speed”. If participants moved their hand in a wrong direction, an error message appeared on the screen for 3 s. After that, they had to try again. The 3 s delay was introduced to discourage participants from randomly trying out directions. RT was computed as the time between the onset of the respective target arrow and the time the participant had moved the plate and handle to one of the four end positions, thereby closing one of the four switches. In trials where the participant had moved the handle to an end position before the respective target arrow appeared, RT was computed as zero. That is, anticipations that occurred earlier than the arrow were registered as RT = 0 ms.

In each run, the arrow sequences were constructed so that each arrow direction appeared three times, once followed by the same direction, once followed by each of the two orthogonal directions, but never followed by the opposite direction. For example, an upward-pointing arrow was followed once by another upward-pointing arrow, once by a leftward-pointing arrow, once by a rightward-pointing arrow, but never by a downward-pointing arrow. Thus, all directions and all transitions between successive directions were balanced within the sequence. A transition between opposite directions was avoided in order to accommodate the maze context: when following a route, we normally do not walk back in the direction from which we have just come.

Sequence learning was assessed by 20 practice runs and one subsequent transfer run. Each practice run presented the same sequence of 12 arrows, and participants therefore could acquire knowledge about this sequence throughout the practice runs. The transfer run presented a different sequence of 12 arrows; participants were not informed about the sequence change, but they could notice it early on, since already the first or the second arrow direction in the transfer run (depending on the sequence) differed from the corresponding arrow direction in the practice runs.

Each participant underwent 20 practice runs and one transfer run in one context (no maze, horizontal maze or vertical maze, see below) and, after a short rest break, 20 practice runs and one transfer run in another context. This allowed us to evaluate the effects of context as a within-subject variable (see below).

### Design and data analysis

We conducted a pilot study and a subsequent main study. In the pilot study, each participant was tested once with the horizontal maze for context, and once with the vertical maze for context. The order of testing (horizontal maze first, vertical maze first) and the response plane (horizontal, vertical) were balanced across participants. If we code the arrow sequences with left as 1, forward (or up) as 2, right as 3, and backward (or down) as 4, then the two sequences were: 3, 2, 2, 1, 4, 4, 1, 1, 2, 3, 3, 4 as well as 4, 1, 2, 2, 3, 3, 2, 1, 1, 4, 3, 4. One arrow sequence was used for the 20 practice runs both of the horizontal maze and of the vertical maze, and the other sequence was used for the transfer run both of the horizontal and of the vertical maze.

In the main study, each participant was tested once with a maze for context, and one without a maze. When the maze was presented, it was oriented horizontally for one half of the participants and vertically for the other half. The order of testing (with maze first, without maze first) and the response plane (same as maze, orthogonal to maze) were balanced across participants. Four different arrow sequences were used (i.e., two additional sequences compared to the pilot study): one for the practice runs with a maze, another for the transfer run with a maze, a third for the practice runs without a maze, and a fourth for the transfer run without a maze (i.e., the additional sequences were 1, 2, 2, 3, 3, 2, 1, 1, 4, 4, 3, 4 as well as 3, 3, 2, 2, 1, 2, 3, 4, 4, 1, 1, 4). Unlike in the pilot study, therefore, participants had to learn a different (but statistically comparable) sequence with the maze and without the maze, so as to avoid any potential carryover learning effects that could have occurred in the pilot study.

In both the pilot study and the main study, responses in a wrong direction were excluded from analysis (about 1% of responses). Responses to the first arrow in a run were excluded as well, to avoid a task adjustment bias (Sleezer & Hayden, 2016). Among the remaining responses, those with RTs of less than 100 ms were classified as anticipations, and those with RTs outside a window of ± 3.29 standard deviations about the mean were classified as outliers (Tabachnick & Fidell, 2001). One dependent variable for statistical analysis was RT, the mean RT per run of those responses that were neither anticipations nor outliers. A second dependent variable was a composite score (CS) of RT and the number of anticipations per run. We decided to include CS since sequence learning might manifest both by faster reactions and by more anticipations. In analogy to composite scores often used in cognitive and motor research (e.g. Liesefeld et al., 2015; Schoene et al., 2013), we defined

1 $${\text{CS}}\,{ = }\,{\text{z}}\left( {{\text{RT}}} \right) - {\text{z}}\left( {\text{number of anticipations}} \right)$$

where z refers to a z-transformation based on the mean and standard deviation of all responses analyzed in a study. Note that lower CS, just like shorter RT, denotes better performance.

To assess the general benefits of practice, we compared participants’ performance across the 20 practice runs by analyses of variance (ANOVAs). RT was the dependent variable in one ANOVA, and CS was the dependent variable in another ANOVA. Context and Run served as independent variables with repeated measures in both ANOVAs. The levels of Context were ‘horizontal’ and ‘vertical’ in the pilot study, and were ‘with maze’ and ‘without maze’ in the main study. The levels of Run were 1, 2, …, 20 in both studies.

To assess the magnitude of sequence-specific learning, we compared participants’ performance on the last practice run (Run 20) to that on the transfer run (Run 21, where the arrow sequence had a similar statistical structure but different sequential transitions). Hence, performance decrements on the transfer run was taken as an indirect measure of sequence-specific learning. This was done by the same ANOVA models as above, except that Run now had the levels 20 and 21.

## Results

### Pilot study

Response errors, where the hand moved in a direction other than the arrow direction, occurred in only 1.07% of responses; the incidence of those errors did not differ between the horizontal and the vertical maze in a test of proportions (*p* = 0.645).

**Fig. 2:**
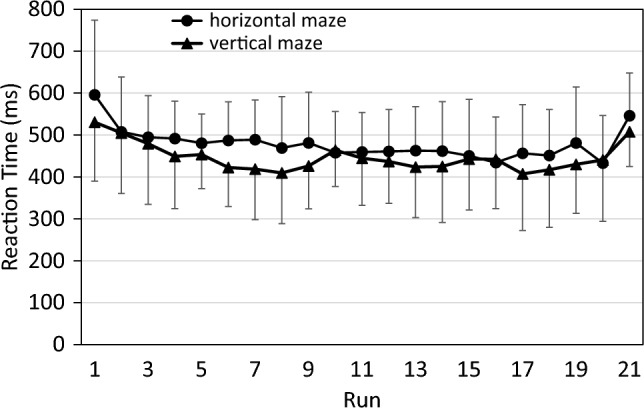
Reaction time (excluding anticipations) across runs in both maze contexts of the pilot study. Error bars represent standard deviations

Figure 2 shows that RTs decreased over the 20 practice runs and that RTs increased again in the transfer run. Note that five of the 24 participants had to be excluded from these analyses because of missing data: all their responses on one or more runs were anticipations, and thus no RTs could be calculated.

An ANOVA of practice benefits yielded a significant effect of Run (*F*(19,342) = 9.19; *p* < 0.001; partial η^2^ = 0.33), but not of Context (*F*(1,18) = 2.30; *p* = 0.147; partial η^2^ = 0.11). The interaction was not significant either (*F*(19,342) = 0.84; *p* = 0.653; partial η^2^ = 0.04). An ANOVA of sequence-specific learning, focusing on the negative transfer effect with Run 21, also yielded a significant effect of Run (*F*(1,18) = 12.74; *p* = 0.002; partial η^2^ = 0.41), but not of Context (*F*(1,18) = 0.04; *p* = 0.839; partial η^2^ < 0.01) or the interaction of Run and Context (*F*(1,18) = 2.55; *p* = 0.128; partial η^2^ = 0.12).

**Fig. 3:**
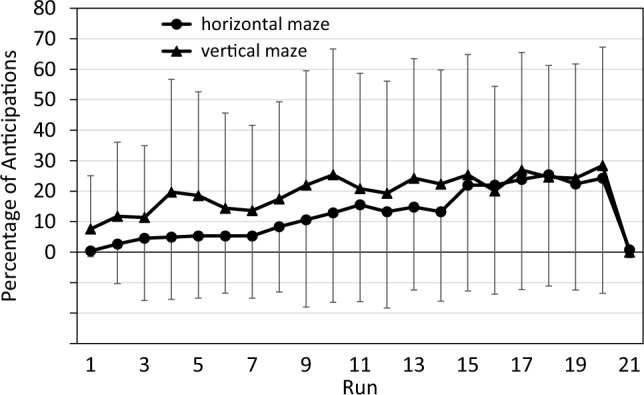
Percentage of anticipations across runs in both maze contexts of the pilot study. Error bars represent standard deviations

Notably, the data shown in Fig. 2 do not capture the full effect of runs and contexts on performance since they disregard those performance changes which manifest not as shorter RTs, but rather as more anticipations. Specifically, some participants produced a mix of anticipations and valid RTs, but only the latter were included in the above analyses, which thus underestimates their actual performance level. Furthermore, some participants produced only anticipations during some of their later runs and were therefore excluded from above analyses, which underestimates the performance level of the participant group as a whole. Figure 3 illustrates that anticipations followed a reciprocal pattern to RT: the percentage of anticipations increased throughout the 20 practice runs, and decreased sharply in the transfer run.

**Fig. 4:**
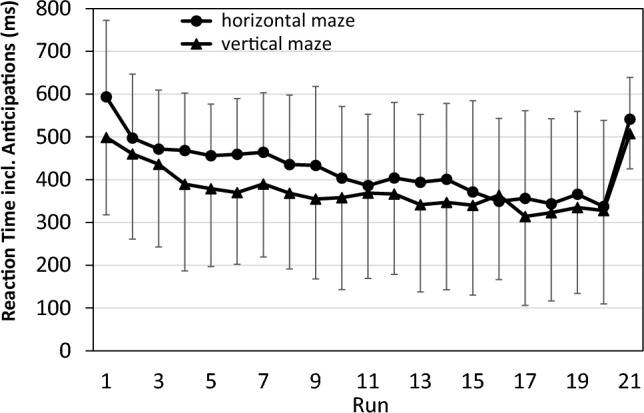
Reaction time in the pilot study when anticipations are treated as reaction times. Error bars represent standard deviations

Figure 4 illustrates one approach to take anticipations into account: they are treated as if they were extremely short RTs and are averaged in each run along with the regular RTs. That is, our experimental software started to capture responses at the time of arrow appearance, such that anticipations that occurred earlier than the arrow were registered as RT = 0 ms.

**Fig. 5:**
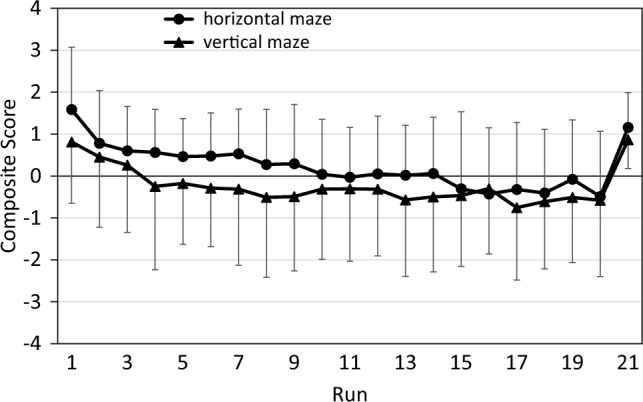
Composite scores in the pilot study, combining RT and anticipations. error bars represent standard deviations

Compared to Fig. 2, the decrease over practice runs and the increase in the transfer run are now more pronounced, and an advantage of the vertical over the horizontal maze context is now more easily discernible. However, the data in Fig. 4 are not suitable for analysis by parametric tests since they are not on an interval scale; for example, the difference between the scores of 200 ms and 100 is not equivalent to the difference between the scores of 100 ms and 0 ms. We therefore rather used CS for statistical analyses (see Fig. 5).

Figure 5 depicts the pattern of CS scores. The pattern is quite similar to that in Fig. 4, except that the difference between the two maze contexts now is even more pronounced. An ANOVA of practice benefits yielded a significant effect not only of Run (*F*(19,437) = 13.08; *p* < 0.001; partial η^2^ = 0.36), but also of Context (*F*(1,23) = 6.86; *p* = 0.015; partial η^2^ = 0.23): CS was lower with the vertical than with the horizontal maze. The interaction of Run and Context was not significant (*F*(19,437) = 1.35; *p* = 0.148; partial η^2^ = 0.06). An ANOVA of sequence-specific learning again yielded a significant effect of Run (*F*(1,23) = 24.27; *p* < 0.001; partial η^2^ = 0.51). However, both the main effect of Context (*F*(1,23) = 1.79; *p* = 0.193; partial η^2^ = 0.07) and the interaction of Run and Context was not significant (*F*(1,23) = 0.62; *p* = 0.440; partial η^2^ = 0.03).

In sum, in the pilot study we found general practice benefits for both the vertical and the horizontal context, but these benefits occurred at the same rate in both contexts. We also found substantial sequence-specific learning effects in both contexts, again of similar size for both contexts. Overall performance was somewhat better for the vertical maze in the practice phase, but this effect did not change across runs and disappeared at the test of sequence-specific learning. The effect was thus neither related to practice benefits nor to sequence-specific learning. Therefore, we concluded from the pilot study that sequence learning was similar for vertical and horizontal maze contexts. Hence, for the main study, we compared sequence learning with a maze context (either vertical or horizontal) with that without a maze context.

### Main study

**Fig. 6:**
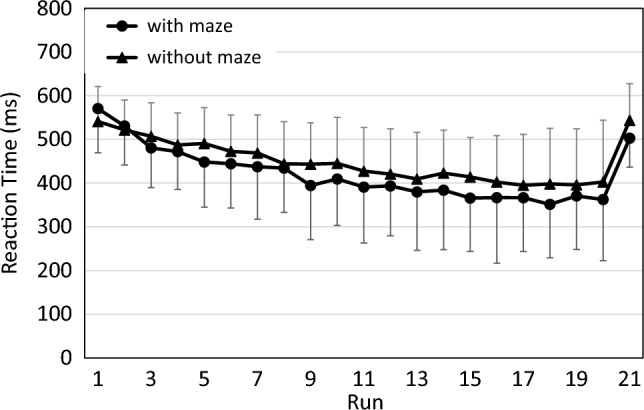
Reaction time (excluding anticipations) across runs in both contexts of the main study. Error bars represent standard deviations

Response errors occurred in only 0.68% of responses and their incidence did not differ with vs. without the maze in a test of proportions (*p* = 0.566). As in the pilot study, RT decreased from the first to the last practice run and increased again in the transfer run, as shown in Fig. 6.

**Fig. 7:**
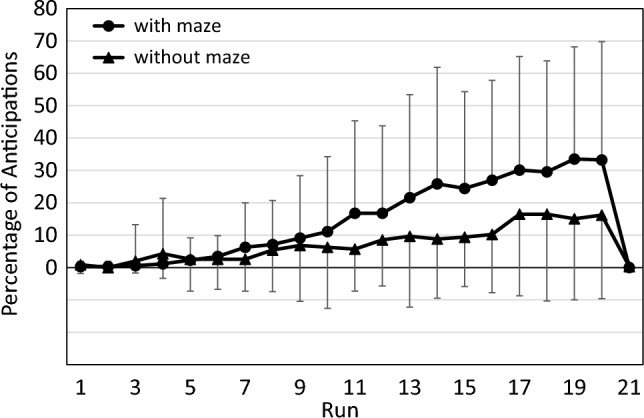
Percentage of anticipations across runs in both contexts of the main study. Error bars represent standard deviations

An ANOVA of practice benefits yielded a significant effect of Run (*F*(19,494) = 38.82; *p* < 0.001; partial η^2^ = 0.60). However, the effect of Context (*F*(1,26) = 1.36; *p* = 0.254; partial η^2^ = 0.05) and of the interaction of Run and Context (*F*(19,494) = 1.43; *p* = 0.105; partial η^2^ = 0.05) was not significant. The subsequent ANOVA for sequence-specific learning yielded a significant effect of Run (*F*(1,28) = 41.20; *p* < 0.001; partial η^2^ = 0.60), and of Context (*F*(1,28) = 8.89; *p* = 0.006; partial η^2^ = 0.24): RT in Run 20 and 21 was shorter with the maze context than without maze context. The interaction of Run and Context was not significant (*F*(1,28) = 0.02; *p* = 0.876; partial η^2^ < 0.01). Note that five participants had to be excluded from the analysis of practice benefits and three from the analysis of sequence learning, because all their responses on one or more runs were anticipations (see Fig. 7).

**Fig. 8:**
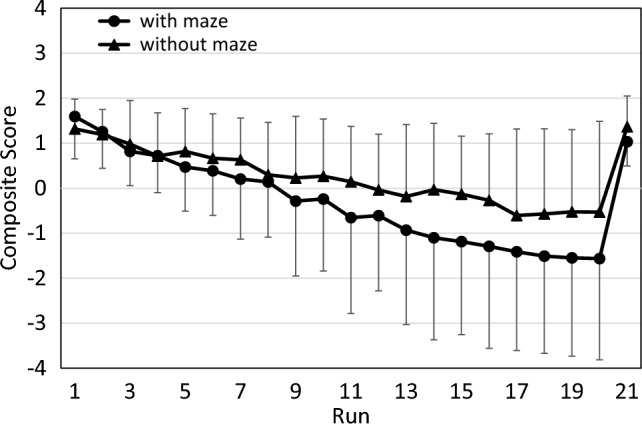
Composite scores in the main study, combining RT and anticipations. error bars represent standard deviations

As in the pilot study, anticipations followed a reciprocal pattern to RT. They increased throughout the 20 practice runs, and decreased sharply in the transfer run. Like in the pilot study, we did not analyze the number of anticipations separately but instead focused on CS, which takes RT and anticipations jointly into account (see Fig. 8).

Figure 8 shows that CS followed a similar pattern as RT, with two notable exceptions. First, the difference between contexts increased substantially during the later practice runs. Second, the increase from the last practice run to the transfer run was more pronounced with than without a maze. Accordingly, an ANOVA of practice benefits yielded a significant effect of Run (*F*(19,589) = 35.01; *p* < 0.001; partial η^2^ = 0.53), of Context (*F*(1,31) = 4.70; *p* = 0.038; partial η^2^ = 0.13), and of the interaction of Run and Context (*F*(19,589) = 3.99; *p* < 0.001; partial η^2^ = 0.11). Furthermore, an ANOVA of sequence-specific learning likewise yielded a significant effect of Run (*F*(1,31) = 42.12; *p* < 0.001; partial η^2^ = 0.50) and Context (*F*(1,31) = 14.06; *p* < 0.001; partial η^2^ = 0.31). Again, the interaction of Run and Context was significant, too (*F*(1,31) = 5.37; *p* = 0.027; partial η^2^ = 0.15).

To summarize the results of the main study, there were strong general practice benefits and sequence-specific learning effects with the arrow sequence alone, without the maze context. Importantly, however, both effects were significantly stronger with the maze context.

## Discussion

Two studies examined the role of meaningful contexts for sequence learning, using a serial RT task. In this task, participants saw a sequence of arrows pointing in one of four directions, and each arrow direction was mapped onto a spatially compatible response direction, executed by moving a lever. A sequence of 12 arrows comprised a run. Participants performed 20 runs with the same arrow sequence to enable practice and sequence-specific learning, and a transfer run with a different arrow sequence to assess the magnitude of sequence-specific learning. A meaningful context was introduced by presenting the arrows not against a homogeneous background, but rather as indicators of a route through a maze. The pilot study compared participants’ performance with a horizontally oriented maze context to that with a vertically oriented maze context, while the main study compared performance with and without a maze context. In either case, these comparisons were based on a within-subject design.

Inspection of the data revealed a decrease of RT from Run 1 to Run 20, which was paralleled by an increase in the rate of anticipations. We attribute the unusually high anticipation rate to the fact that, necessitated by a realistic speed of transport from one intersection to the next, the response-to-stimulus interval (RSI) was quite long; our RSI = 2.3 s was much longer than in typical studies on sequence learning, where RSI ≈ 500 ms. Future research could reduce the anticipation rate by increasing the speed of transport, or by sanctioning anticipations, but either of these two approaches would reduce the ecological validity of the maze context and thus might also reduce the magnitude of the context effects reported here.

We reasoned that participants improved their performance not only by reacting faster, but also by switching from reactions to anticipations, such that an analysis of RTs alone cannot capture the full extent of practice benefits. Moreover, some participants produced exclusively anticipations on their later practice runs and therefore had to be excluded from an analysis of reaction times. We therefore decided to analyze not only the RTs, but also constructed a compound score that integrates RT and anticipation rates. In this way, we sought to capture the practice and sequential learning effects in the most comprehensive fashion, even if we deviate from standard data analyses with the serial RT task (see Schwarb & Schumacher, 2012, for a review).

### Key findings

The primary purpose of the pilot study was to establish a novel experimental paradigm, consisting of a serial RT task embedded in a maze context. The data indicated robust general practice benefits and sequence-specific learning effects. A secondary purpose was to explore possible differences between a horizontal and a vertical maze: based on the fact that we usually move through a horizontally oriented environment, we wondered whether a horizontal maze might provide a more effective context than a vertical maze. Yet, we found no support for such a difference, since practice and sequence-specific learning effects were comparable with both mazes. However, we unexpectedly yielded some evidence for a better overall performance with the vertical maze, possibly because the unusual vertical architecture increased the participants’ level of alertness. Yet, this presumed increase was only transient, since the vertical maze advantage dissipated later in practice and on transfer, so that the data do not allow claims about performance differences with vertical vs. horizontal mazes.

The primary purpose of the main study was to compare learning with and without a maze context. Although maze orientation had no effect on practice and sequence-specific learning in the pilot study, we still decided to vary the orientation in the main study to obtain a more generalized result (averaging across the two orientations). We further decided to use different arrow sequences in runs with and without a maze to prevent carry-over of learning. The data again documented robust general practice benefits and sequence-specific learning effects. Moreover, CS revealed a substantial influence of the maze context on learning: both general practice benefits and sequence-specific learning effects were larger with the maze than without the maze. This suggests that the maze context did not degrade sequence learning by means of resource competition (overview e.g. in Schumacher & Schwarb, 2009), but rather enhanced sequence learning.

Note that our assessment of sequence learning also takes into account anticipatory responses, which can be taken as indication of explicit predictions (e.g., Nissen & Bullemer, 1987), so that our data most likely do not represent a case of implicit learning in the sense of unconscious, non-verbalizable learning (Dienes & Berry, 1997; Esser et al., 2022). Note that the empirical distinction of implicit and explicit learning within the same task is methodologically very intricate and complex (see Shanks & St. John, 1994, for a discussion) and it has been argued that the difference may be more quantitative than qualitative (e.g., Cleeremans & Jiménez, 2002). In fact, the empirical learning effects in terms of the indirect RT measure often correlates very highly with the amount of explicit knowledge (e.g., Koch, 2007; Zirngibl & Koch, 2002). Yet, because our data do not allow us to quantify the contribution of implicit sequence learning in the absence of any explicit learning, we remain rather agnostic with respect to this distinction, even though we believe that presumably many or even most participants developed explicit sequence knowledge at least to some degree.

### The influence of environmental context on sequence learning

We observed successful sequence-specific learning effects when arrows were presented against a homogeneous background, which documents that the basic mechanisms of sequence learning (see Schwarb & Schumacher, 2012) can be studied fruitfully using the arrow stimuli, stimulus sequences, response-to-stimulus intervals, and response types that we implemented. Notably, the present novel experimental paradigm enabled us to produce different response consequences in the given maze contexts even though the basic stimulus sequence is very similar and the corresponding response sequence is identical across learning conditions. Importantly, practice benefits and sequence-specific learning effects were larger when arrows were presented not against a homogeneous background, but rather as waymarks in a maze, irrespective of the horizontal or vertical orientation of that maze.

It might seem surprising at a first glance that adding nominally task-irrelevant environmental cues to the task, such as a horizontal or vertical maze, served to facilitate learning. We attribute this facilitation to a more enriched mental representation of the arrow sequence, brought about by contextual associations (Godden & Baddeley, 1975; Yonelinas et al., 2019) between the arrows and the maze. We can envision two mutually not exclusive possibilities of how this facilitation came about. First, the maze context might encourage a process of chunking of stimuli or responses into hierarchically organized subsequences, thus allowing a more efficient sequence representation (Jiménez et al., 2011; Koch, 2007; Schwarb & Schumacher, 2012). We can only speculate about the factors that might trigger chunking, such as the implied biomechanical (Kim et al., 2020) and cognitive demands of changing one’s direction of walking. Future studies could be designed specifically to detect the formation of such chunks, and thus to scrutinize this first possibility. Second, the maze context might encourage the formation of a spatial representation, or ‘cognitive map’ (O'Keefe & Nadel, 1978; Tolman, 1948), of the maze and of an arrow-marked route through it. Hence rather than learning a sequence of directions, participants might have learned the spatial layout of a route and/or the anticipated change in the environment based on a process of anticipation the consequences of one’s own actions (e.g., response-effect learning; Ziessler & Nattkemper, 2001; see Shin et al., 2010, for a general review of “ideomotor” approaches). In any case, it seems likely that environmental maze contexts enrich sequential action planning and that chunking and/or map formation seemed to be similarly efficient in the vertical and in the horizontal plane.

### Implications for our understanding of spatial representations

If one subscribes to the second above interpretation, according to which the maze in our studies encouraged the spatial representation of an arrow-marked route, then the present data may be relevant for the understanding of the basic mechanisms involved in spatial representations. While some earlier research suggested that buildings are mentally represented as a stack of horizontal layers (Jeffrey et al., 2013; Montello & Pick, 1993; Thibault et al., 2013; Zwergal et al., 2016), other work indicated that this preference for horizontal layers is due to a physical rather than to a cognitive constraint: the floors and ceilings of most buildings obstruct the integration of spatial information in the vertical plane, and when this physical constraint is avoided, participants no longer exhibit a preference for horizontal layers (Bock et al., 2020; Ishikawa & Montello, 2006; Lu & Ye, 2019; Tlauka et al., 2007). The second interpretation of the present findings is in agreement with this view, since it suggests that learning and using the spatial representation of a vertically oriented route is not less efficient than that of a horizontally oriented route if exploration in the vertical plane is not more obstructed than that in the horizontal plane. Yet we acknowledge that our data, from the pilot study, are not conclusive enough to derive strong conclusions about different representations of vertical vs. horizontal spaces.

## Conclusion

In sum, the present findings bridge a gap between research on sequence learning and on wayfinding, suggesting that the underlying mechanisms are not isolated cognitive functions and that studying their interaction can reveal new insights. Our data suggest that environmental context cues allow the formation of a more abstract, map-like spatial representation of the required action sequence. This richer representation may facilitate sequence learning relative to a “pure” condition in which target stimuli and response occur in isolation, without a maze context. Using virtual maze contexts for studying sequence learning offers interesting new methodological approaches to varying action effects in otherwise unchanged target and response sequences in order to examine factors influential for facilitating learning in complex environments.

## Data Availability

The datasets generated during and/or analysed during the current study are available from the corresponding author on reasonable request.
